# Is N-methylacetazolamide a possible new therapy against ischemia-reperfusion injury?

**DOI:** 10.3389/fphar.2023.1223132

**Published:** 2023-08-10

**Authors:** Alejandro Ciocci Pardo, Leandro A. Díaz Zegarra, Luisa F. González Arbeláez, Ernesto A. Aiello, Susana M. Mosca

**Affiliations:** Centro de Investigaciones Cardiovasculares “Dr Horacio E Cingolani”, CCT-CONICET, Facultad de Ciencias Médicas, Universidad Nacional de La Plata. La Plata, Buenos Aires, Argentina

**Keywords:** N-methylacetazolamide, myocardial ischaemia, reperfusion injury, calcium, L-type Ca^2+^ channel (CaL), cardioprotection

## Abstract

The increase of intracellular Ca^2+^ concentration, produced principally by its influx through the L-type Ca^2+^ channels, is one of the major contributors to the ischemia-reperfusion injury. The inhibition of those channels in different experimental models was effective to ameliorate the post-ischemic damage. However, at a clinical level, the results were contradictory. Recent results of our group obtained in an ¨*ex vivo*¨ heart model demonstrated that a chemical derived from acetazolamide, the N-methylacetazolamide (NMA) protected the heart against ischemia-reperfusion injury, diminishing the infarct size and improving the post-ischemic recovery of myocardial function and mitochondrial dynamic. A significant inhibitory action on L-type Ca^2+^ channels was also detected after NMA treatment, suggesting this action as responsible for the beneficial effects on myocardium exerted by this compound. Although these results were promising, the effectiveness of NMA in the treatment of ischemic heart disease in humans as well as the advantages or disadvantages in comparison to the classic calcium antagonists needs to be investigated.

## Introduction

Ischemic heart disease (IHD) is one of the most frequent causes of heart failure and remains the leading reason of mortality worldwide (Bauersachs et al., 2019). IHD is normally attributed to coronary artery disease leading to a diminution of blood supply, myocyte death and contractile impairment (Severino et al., 2020). Although reperfusion appears as the best strategy to attenuate the ischemic damage, it produces an additional damage called reperfusion injury. Several interventions and treatments have been developed to attenuate the ischemia-reperfusion injury. In this sense, it was previously described that ischemic pre-and postconditioning confers cardioprotection through the activation of several protective mechanisms by the application of short periods of ischemia-reperfusion, before or after the main insult, respectively (Heusch, 2015; Donato et al., 2017; Wu et al., 2021). Although numerous factors have been implicated in the ischemia-reperfusion injury, Ca^2+^ overload appears to play a crucial role (Dibb et al., 2007; Murphy and Steenbergen, 2008; Kalogeris et al., 2016; Pittas et al., 2018).

## Ca^2+^ overload: role of l-type Ca^2+^ channel

In cardiac muscle, membrane depolarization produces the influx of extracellular Ca^2+^ through the cardiac α1 subunit of voltage-gated L-type Ca^2+^ channel (CaC) which activates ryanodine receptor 2 (RyR2). Ca^2+^ release triggers shortening of the contractile unit, the sarcomere, resulting in the force generation. Muscle relaxation occurs when Ca^2+^ is removed from the contractile unit through the combined action of Ca^2+^ pumps and Na^+^/Ca^2+^ exchangers (Barry and Bridge, 1993; Dibb et al., 2007).

The purified CaC contains five subunits, the principal or pore-forming subunit, α_1_ and different auxiliary subunits, α_2_, β, δ, and γ. The auxiliary subunits are non-covalently linked to the α_1_ subunit, modulating the biophysical properties and trafficking of the α_1_ subunit onto the membrane (Catterall, 1995; Striessnig et al., 2015). The α_1_ subunit corresponds to the pore-forming segment of CaC that allows the passage of Ca^2+^ ions and is composed of approximately 2000 amino acids. The other components serve as auxiliary subunits modifying the channel function.

Ca^2+^ homeostasis is particularly important for myocardial cell structure and function. During ischemia-reperfusion this process is altered and the intracellular Ca^2+^ concentration increases generating the previously mentioned Ca^2+^ overload (Smith and Eisner, 2019; Wang et al., 2020). Therefore, the prevention and or the treatment of Ca^2+^ overload can protect cardiac myocytes against ischemia-reperfusion injury (Han et al., 2019; Wang et al., 2020). One useful approach to prevent or treat Ca^2+^ overload is the sarcolemmal Ca^2+^ channels blockers, demonstrating that CaC is an important target to protect the heart against ischemia-reperfusion injury (Talukder et al., 2009). These drugs such as dihydropyridines (DHPs) or phenylalkylamines, bind to a region close to the pore (α1-subunit) decreasing the Ca^2+^ entry to the cell.

Previous studies from our and other laboratories demonstrated in ¨*ex vivo*¨ experimental models the beneficial effects of Ca^2+^ channels blockers on the hearts during reperfusion (Moreyra et al., 1994; Chiappe de Cingolani et al., 1996; Simonovic and Jeremic, 2017) showing a reduction of infarct size and the postischemic contractile dysfunction. At a clinical level a reduction in myocardial oxygen consumption due to negative inotropic and chronotropic effects of CaC blockers were referred as anti-ischemic effects of these compounds (Gross et al., 1999). However, the use of some of them in patients did not modify the survival and post-infarct cellular injury (Elliott and Ram, 2011; Sueta et al., 2017). In other words, the clinical use of traditional CaC blockers in myocardial infarct is still in dispute because of their marked hemodynamic effects. Therefore, the exploration of effective therapeutic strategies against these cardiovascular disorders is still an essential research direction.

## N-methylacetazolamide (NMA)

N-methylacetazolamide (NMA) belongs to a group of chemicals derived from the carbonic anhydrase (CA) inhibitor acetazolamide. In this case, a methyl group substituted one H^+^ in the sulfonamide moiety of acetazolamide. This substitution results in an approximately 200-fold decrease in binding affinity to CA maintaining the physical–chemical properties of acetazolamide (Maren, 1956; Teppema1 and Swenson, 2015).

The lack of inhibitory action of NMA on CA was revealed in our experiments on isolated papillary muscles subjected to an acid load, which showed that this drug did not contribute to the H^+^ efflux or the intracellular pH recovery (Ciocci Pardo et al., 2022).

## Cardiac effects

We recently demonstrated the beneficial effects of NMA on the alterations subsequent to ischemia and reperfusion in the isolated rat heart (Ciocci Pardo et al., 2022). Our experiments showed that the treatment of hearts during the first 10 min of reperfusion with NMA 5 μM was able to decrease the infarct size (measured by TTC staining technique and expressed as percentage of risk area) produced by 30 min of global ischemia and 60 min of reperfusion. Thus, while in untreated hearts an infarction of approximately 35% was detected, in those treated with NMA this value was lower, of approximately 20%. NMA also significantly improved the postischemic myocardial function. An increase of systolic function (assessed by left ventricular developed pressure, LVDP) and a decrease diastolic stiffness (assessed by left ventricular end diastolic, LVEDP) were some beneficial effects observed in NMA treated hearts. That is, acute treatment with NMA was efficient to decrease the cell death and myocardial dysfunction following to ischemia-reperfusion.

## Molecular mechanisms

**FIGURE 1:**
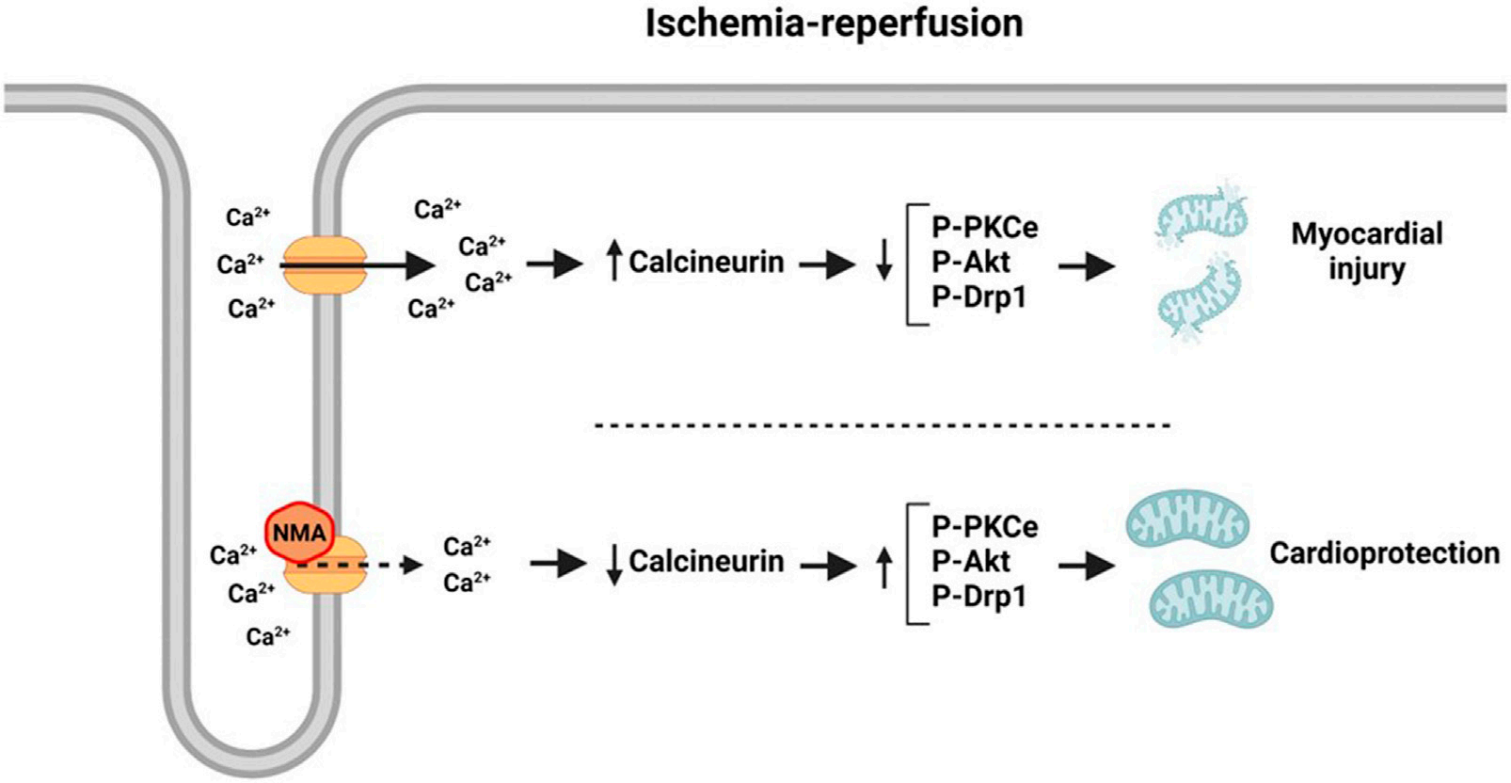
Schematic representation of L-type Ca^2+^ channel structure and its typical current in untreated and NMA treated hearts.

It was previously documented that the increase in intracellular Ca^2+^ concentration induced by hypoxia -measured by the use of Ca^2+^sensitive dye fura2-was markedly reduced in pulmonary arterial smooth muscle cells treated with NMA (Shimoda et al., 2007). These authors concluded that this action of NMA was independent of CA inhibition. In isolated myocytes we observed a similar action detecting a decrease of CaC current, measured by patch clamp technique in whole cell configuration, after NMA treatment (Figure 1). The inhibition of CaC current is rapidly turned on, suggesting the direct binding of NMA to the channels. On the other hand, voltage-dependence of activation and inactivation were not affected by NMA. Thus, a direct effect of the compound on the pore without affecting the biophysical properties of the channel is most likely (Ciocci Pardo et al., 2022).

Taking into account these data, our first conclusion was that a diminution of Ca^2+^ overload, probably mediated by a direct binding of NMA to CaC would be a possible mechanism involved in the beneficial effects achieved by NMA on ischemic myocardium. The increase in cytosolic Ca^2+^ during ischemia has been associated with an enhancement of sarcoplasmic reticulum (SR) Ca^2+^ load, which is released at the onset of reperfusion and produces an abrupt rise in cytosolic Ca^2+^ and the consequent decrease in SR Ca^2+^ content and Ca^2+^ transient (Federico et al., 2020). Although the action of NMA on SR was not examined we can speculate that the diminution of Ca^2+^ entry through a direct action of NMA on CaC could attenuate the SR Ca^2+^ release, thus decreasing the intracellular Ca^2+^ concentration, crucial factor of myocardial damage.

The preservation of mitochondrial function is the main mechanism to protect the heart against ischemia-reperfusion injury (Honda et al., 2005; Anzell et al., 2018). Several protein kinases activated immediately prior to or at the time of reperfusion have been implicated in the pathways leading to cardioprotection (Altamirano et al., 2015). Thus, phosphatidylinositol-3-kinase (PI3K/Akt) and PKCε are contributing to myocyte defense against Ca^2+^ overload (Mochly-Rosen et al., 2012; Duan et al., 2015). On the other hand, it was previously demonstrated that calcineurin activation contributes to myocardial postischemic damage probably associated to an increase of Ca^2+^ intracellular concentration via CaC (Lakshmikuttyamma et al., 2003; Tandan et al., 2009).

**FIGURE 2:**
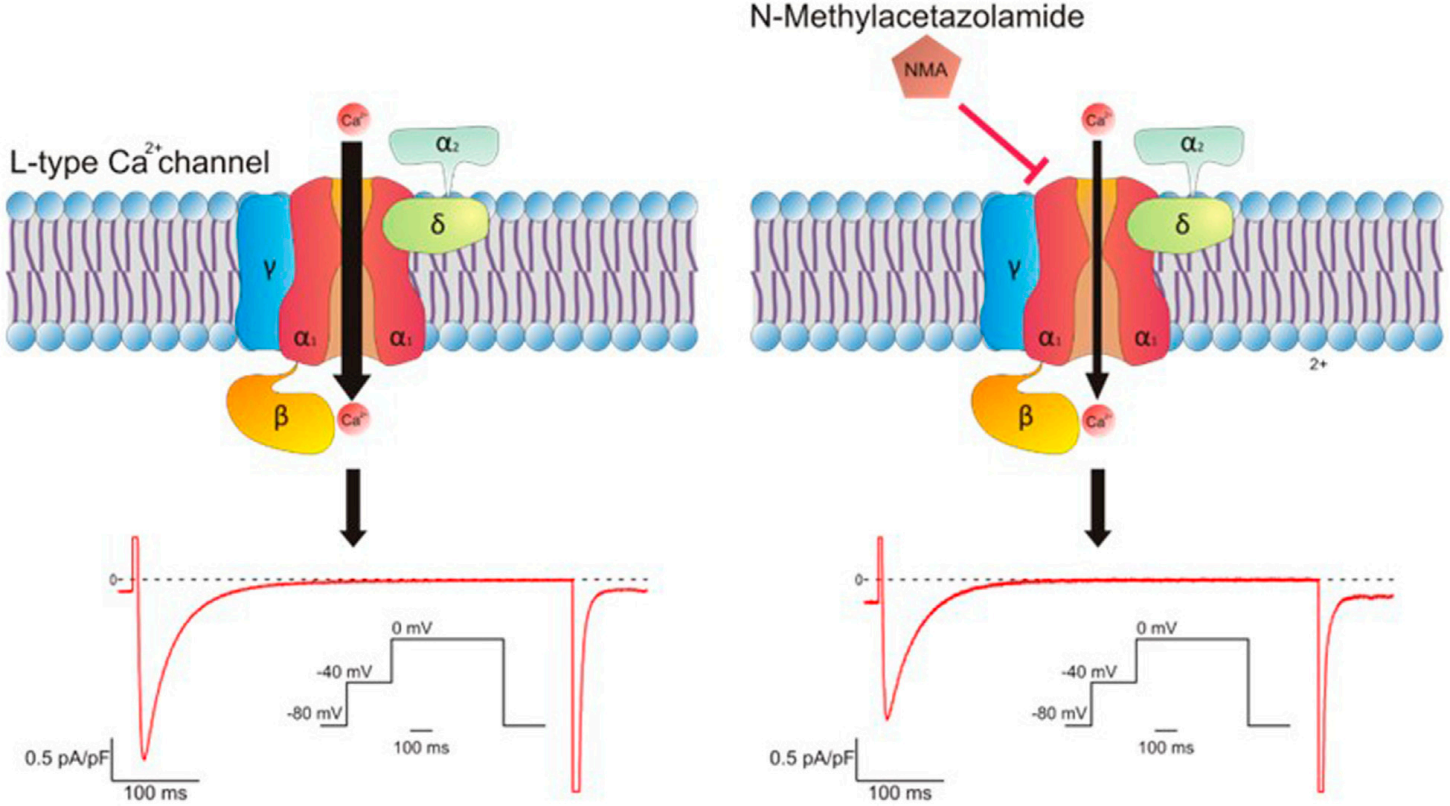
Possible pathway of the cardioprotection NMA-mediated.

Cytosolic Ca^2+^ overload is a key stimulus to open the mitochondrial permeability transition pore (mPTP) and the activation of mitochondrial fragmentation -assessed by dephosphorylation at Ser637Drp1 (a dynamin-related protein-1)- both events occurring during ischemia-reperfusion (Youle and van der Bliek, 2012; Hernandez-Resendiz et al., 2020; Ramachandra et al., 2020). The activation of pathways initiated by the kinases mentioned above and the inactivation of calcineurin have been involved in the prevention or attenuation of those mitochondrial detrimental actions (Baines et al., 2003; Cereghetti et al., 2008; Miyamoto et al., 2008; Maneechote et al., 2017; Wang et al., 2017). Our data demonstrated that the cardioprotection afforded by NMA involve pathways activated by Akt and PKCε and calcineurin inactivation having the mitochondria as a crucial end point. At the level of this organelle, our results also show an increase of Drp1 phosphorylation suggesting that an attenuation of mitochondrial fission could be a possible mechanism involved in the diminution of post-ischemic damage NMA-mediated (Figure 2).

## Conclusion

Taking into account previous data (Ciocci Pardo et al., 2022), we consider that NMA, through the blockade of L-type Ca^2+^ channel in a similar manner to the classic calcium antagonists, represents an attractive alternative to ameliorate the postischemic impairment. According to our experience the main difference between those drugs, such as dihydropyridine (DHP) compounds, and NMA was the administration time; while the classic calcium blockers were given prior to ischemia, NMA was administered at the beginning of reperfusion. This fact is very important, making ¨*a priori*¨ the NMA a superior tool for the treatment of ischemic heart disease in patients. Although this fact is very important, clinical trials will be mandatory to demonstrate the effectiveness of NMA in humans and its advantages or disadvantages in comparison to other calcium antagonists.
